# Ultrasound localization microscopy for evaluation of therapeutic efficacy assessment after transarterial chemoembolization in hepatocellular carcinoma

**DOI:** 10.3389/fonc.2026.1786075

**Published:** 2026-04-28

**Authors:** Ming-yuan Wang, Li Lei, Xu Zhang, Fei Chen, Bao-hua Li, Ya-ning Yao, Long Deng, Jin-duo Zhang, Yue-zhen Sun, Jun-qiang Lei, Shunlin Guo

**Affiliations:** 1 The First School of Clinical Medical, Lanzhou University, Lanzhou, Gansu, China; 2 Department of Ultrasonic Medicine, the First Hospital of Lanzhou University, Lanzhou, Gansu, China; 3 Department of Interventional Medicine, the First Hospital of Lanzhou University, Lanzhou, Gansu, China; 4 Endoscopic Diagnosis and Treatment Center, Gansu Provincial Hospital, Lanzhou, Gansu, China; 5 The Department of General Surgery, the First Hospital of Lanzhou University, Lanzhou, Gansu, China; 6 Tianshui Disease Prevention and Control Center, Tianshui, Gansu, China; 7 Department of Radiology, the First Hospital of Lanzhou University, Lanzhou, Gansu, China; 8 Intelligent Imaging Medical Engineering Research Center of Gansu Province, Lanzhou, Gansu, China; 9 Accurate Image Collaborative Innovation International Science and Technology Cooperation Base of Gansu Province, Lanzhou, Gansu, China; 10 Gansu Province Clinical Research Center for Radiology Imaging, Lanzhou, Gansu, China

**Keywords:** clinical prediction model, hepatocellular carcinoma, multimodal imaging, transarterial chemoembolization, ultrasound localization microscopy

## Abstract

**Objectives:**

The timely and precise evaluation of the efficacy of Transarterial chemoembolization (TACE) in hepatocellular carcinoma (HCC) patients facilitates post preventive and therapeutic therapy. This study aimed to demonstrate the significance of ultrasound localization microscopy (ULM) assessment of TACE.

**Methods:**

A total of 85 HCC patients underwent initial TACE were retrospectively analyzed. The ULM system automatically calculated relevant metrics, including vessel ratio, mean velocity, and perfusion index. A combined evaluation model was constructed by ULM parameters alongside routine laboratory indicators (AFP, WBC, RBC, ALP) selected via Random Forest and Logistic algorithm.

**Results:**

The combined model achieved an Area Under the Curve (AUC) of 0.915 in assessing TACE efficacy. The model demonstrated excellent calibration with a mean absolute error of 0.076 in clinic decision probabilities.

**Conclusion:**

ULM effectively reflects changes in the tumor vasculature in patients with HCC after TACE. Combining with WBC, RBC, ALP, and elevated AFP levels could improve evaluation performance.

## Highlights

- TACE efficacy in hepatocellular carcinoma patients facilitates preventive therapy.
- ULM combined with laboratory markers to effectively assess post-TACE.
- ULM captures changes in tumor vasculature in patients with HCC following TACE.

## Introduction

Globally, hepatocellular carcinoma (HCC) accounts for approximately 865,000 new cases and 758,000 deaths annually (1, 2); China accounts for 42.5% of the new cases and 41.8% of the deaths. Additionally, 84.4% of patients with HCC in China have a Hepatitis B virus (HBV) infection, with an overall 5-year survival rate of 14.1%. Transarterial chemoembolization (TACE) is widely used in the clinical treatment in all pathology (3, 4).

However, owing to factors such as the rich blood supply of HCC and the ease of formation of collateral circulation, the recurrence rate after TACE remains relatively high. Therefore, patients scheduled for TACE should undergo a comprehensive preoperative assessment of their tumor status and liver function. Additionally, regular assessment and follow-up after TACE is essential to effectively diagnose the survival and recurrence of HCC lesions, enabling the timely implementation of treatment strategies to prolong patient survival.

Ultrasonography is the first-line follow-up imaging modality for detecting HCC and hepatic lesions in patients with cirrhosis (5). However, Doppler techniques and contrast-enhanced ultrasonography (CEUS) are limited by physical barriers (6, 7). Although contrast-enhanced magnetic resonance imaging (CE-MRI) and contrast- enhanced computed tomography (CE-CT) are alternative techniques (5, 8, 9). To overcome this limitation, a recent innovation known as CEUS-based ULM has been implemented to address the physical barrier issue. By utilizing contrast agent microbubble tracking technology, ULM enhances the visibility of smaller microvascular structures at the 10 to 100μm scale, dynamically demonstrates tumor blood supply, and quantifies microvascular parameters such as direction, density, and flow velocity. Moreover, the approach is simple and repeatable and presents new clinical opportunities for HCC management.

ULM can be used to evaluate early changes in the microvascular network in response to interventions that alter tissue perfusion, such as TACE for HCC (10). However, there were no relevant studies have been conducted till now.

In summary, this study aimed to evaluate the therapeutic efficacy of TACE in patients with primary HCC using ULM combined with laboratory data to preliminarily explore the value of ULM for assessing the efficacy of TACE.

## Materials and methods

### Study subjects

This retrospective study included 85 patients was conducted in the Ultrasound Department of Lanzhou University and approved by the Ethical Committee of the First Hospital of Lanzhou University (reference number: LDYYLL2025-863) in accordance with the Declaration of Helsinki. Written informed consent was obtained from all patients prior to TACE and ULM.

*Inclusion and exclusion criteria*.

**Figure 1:**
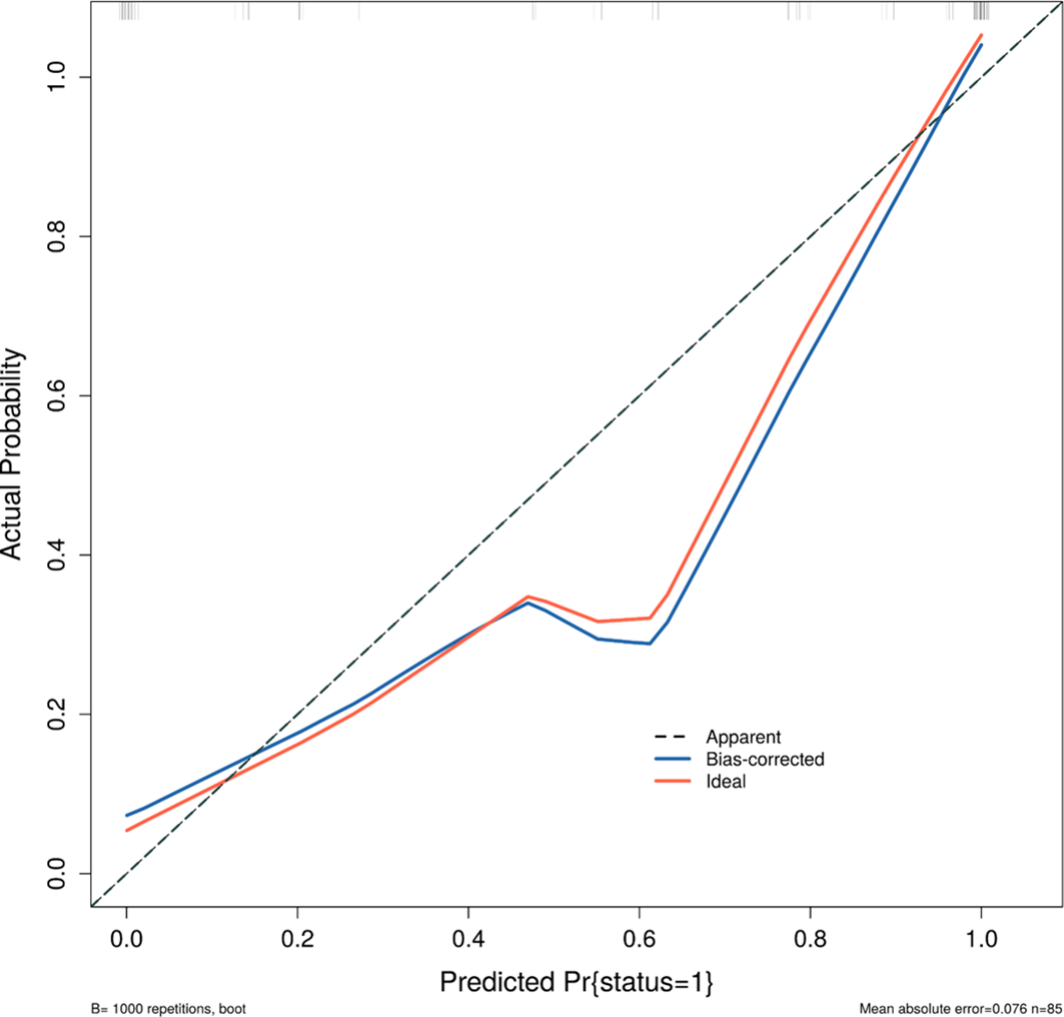
Calibration curve showing the agreement between predicted and observed probabilities.

The patients were diagnosed with primary HCC at the First Hospital of Lanzhou University between September 2024 and September 2025. The inclusion criteria for the prospectively enrolled patients with HCC or lesions were as follows: (1) newly developed, untreated lesions; (2) clear radiographic evidence of HCC; (3) agreement to undergo TACE; and (4) CEUS examination using SonoVue^®^ and ULM video capture. During the study period, 85 patients with radiographic evidence of HCC underwent TACE. The exclusion criteria were as follows: (1) Child-Pugh grade C liver function, (2) patients who had not initially undergone TACE, (3) patients with multiple liver lesions, and (4) patients who had undergone CE-CT before and after TACE for > 6 months. (**Figure 1**) All figures can be observed in **Supplementary Video**.

General baseline demographic and clinical data (including age, sex, lesion size, and location) and laboratory indicators were obtained by searching the electronic medical record system.

### TACE treatment standards

This study was conducted in accordance with the Chinese Clinical Practice Guidelines for Transarterial Chemoembolization of Hepatocellular Carcinoma (2023 edition) and the CIRSE Standards of Practice on Hepatic Transarterial Chemoembolization (11, 12). First, iodized oil emulsions containing chemotherapeutic drugs were used to embolize the distal end of the blood supply artery in liver cancer. Granular embolic agents such as gelatin sponges, blank microspheres, and polyvinyl alcohol were then selected to enhance the embolization effect (12).

### ULM examination method

Patients with HCC were categorized into Complete Response (CR) and Contral group which included Partial Response (PR), Stable Disease (SD), and Progressive Disease (PD) based on comparisons of contrast-enhanced CT images, according to the mRECIST assessment (13–16). (**Table 1**).

**Table 1:** Assessment of target lesion response in mRECIST.

CR	Disappearance of any intratumoral arterial enhancement in all target lesions
PR	At least a 30% decrease in the sum of diameters of viable (enhancement in the arterial phase) target lesions, taking as reference the baseline sum of the diameters of target lesions
SD	Any cases that do not qualify for either partial response or progressive disease
PD	An increase of at least 20% in the sum of the diameters of viable (enhancing) target lesions, taking as reference the smallest sum of the diameters of viable (enhancing) target lesions recorded since treatment started

#### Instruments and equipment

A VINNO ULTIMUS 9E Ultrasound system (VINNO Technology, Suzhou, China) equipped with an S1-8C convex probe (frequency range, 1.5–6.5 MHz) was employed. The system’s quad-core heterogeneous architecture (dual GPUs, CPU, and FPGA) enabled ultrafast plane- wave imaging at 7.2 × conventional frame rates (70–109.5 Hz).

#### ULM examination

**Figure 2:**
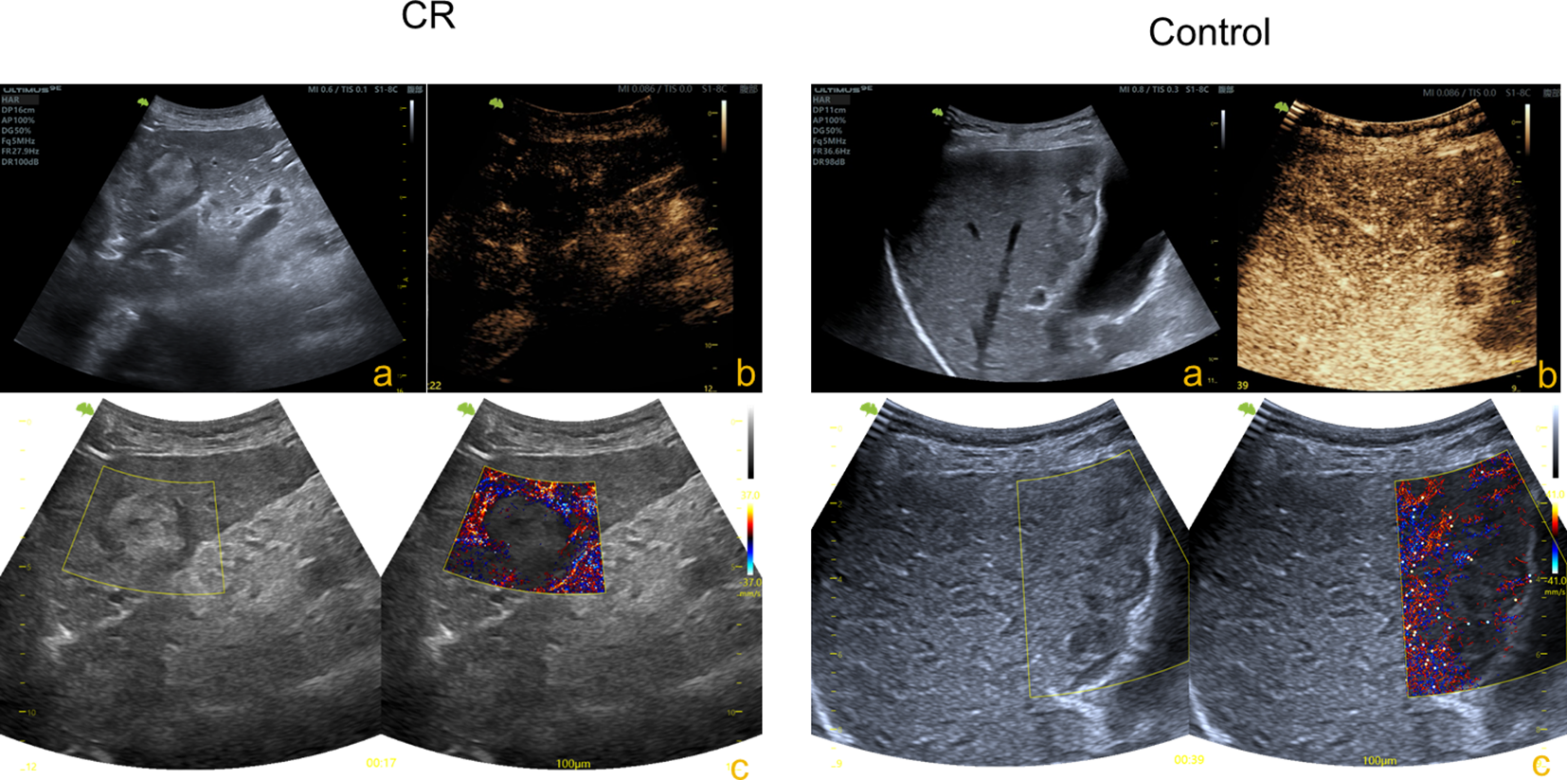
ULM examination in two groups showed different microvascular. **(A)** 2D ultrasound liver examination, localized lesions. **(B)** CEUS of lesions. **(C)** ULM of lesions.

After conventional 2D ultrasound liver examination, lesions were localized (**Figure 2A**). All of the patients underwent CEUS examinations, performed using a real-time, low-mechanical index (0.05–0.08) imaging technique. A bolus injection of 1.0 mL of sulfur hexafluoride microbubbles (SonoVue^®^, Bracco, Milano, Italy) was administered intravenously, immediately followed by a 5.0 mL flush of 0.9% sterile sodium chloride solution. When the injection was completed, a timer and video recordings were started. All CEUS procedures were performed by physicians with at least five years of experience in abdominal CEUS diagnosis. Breath-holding maneuvers were employed to minimize motion artifacts and enhance microbubble-tissue separation. Targeted peak enhancement phases were acquired, in which 10-second sequences (>1000 frames) were recorded for super-resolution processing (**Figure 2B**). The ULM images were generated by the ultrasound system (17) (**Figure 2C**).

### Collection of imaging parameters

**Figure 3:**
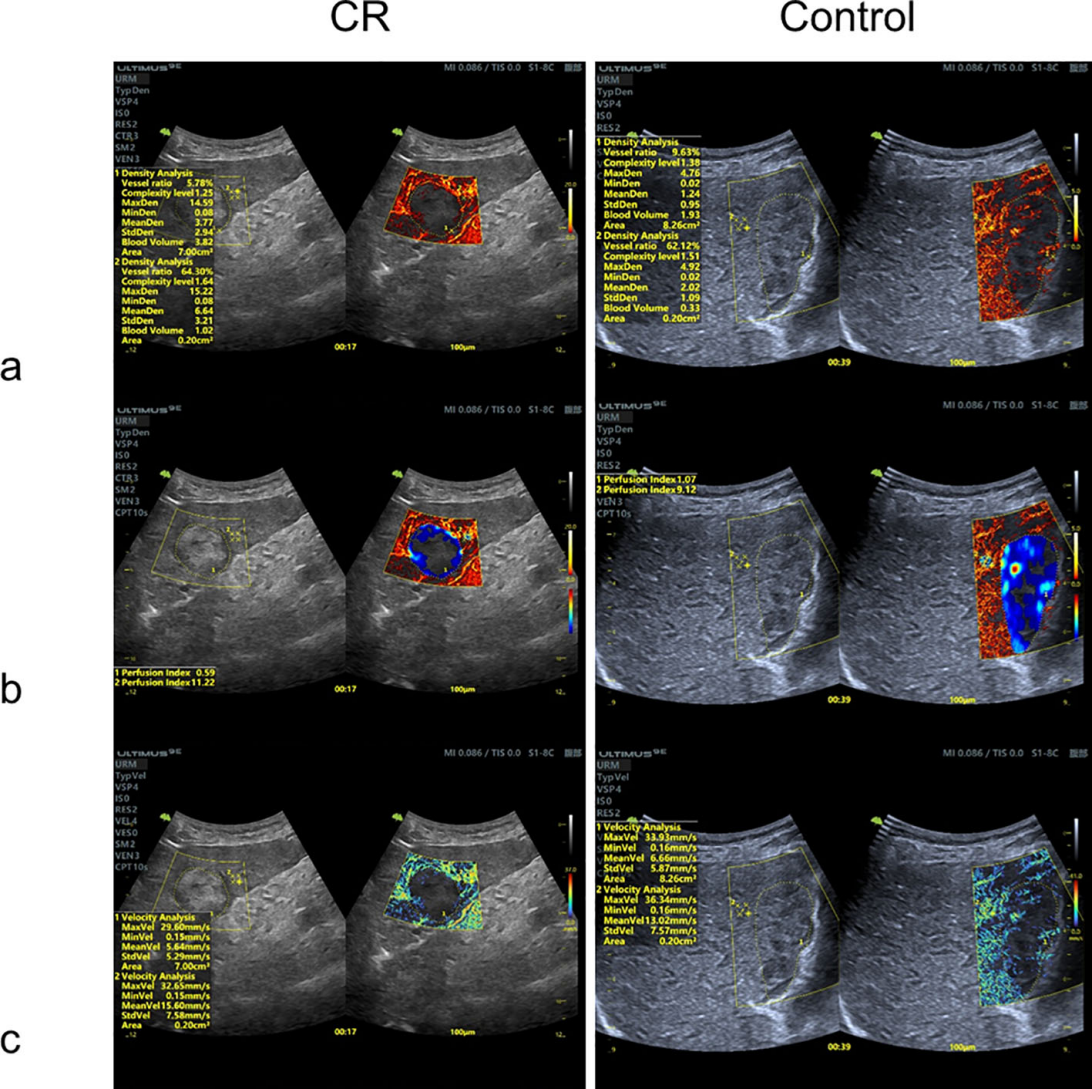
ULM quantitative analysis maps. **(A)** vascular density map; **(B)** vascular perfusion index map; **(C)** hemodynamic velocity map. The quantitative parameters of the lesions and the surrounding normal liver tissue were obtained, as shown in Figures **(A–C)**.

Two ultrasound experts independently delineated the lesion Region of Interest (ROI) and normal liver tissue of 0.2 cm2, located 0.5 cm away from the lesion in the ULM. The raw data underwent microbubble localization via centroid detection, motion tracking, and trajectory mapping to reconstruct microvasculature at sub-100 μm resolution. The integrated super- resolution software (Vinno Technology) was utilized to generate maps of vascular density, vascular perfusion index, and hemodynamic velocity for subsequent quantitative analysis (**Figures 3A–C**). The system then automatically calculated the vessel ratio, mean vessel density, perfusion index, and mean velocity within the lesion and surrounding liver tissue. The assessed ULM features included lesion location, size, vessel ratio, mean vessel density, perfusion index, and mean velocity (17, 18).

Vessel ratio: Vessel Density ROI Area/ROI Area.

Mean Vessel density: Sum of Vessel Density in the Vessel Density Region of ROI/Pixel Count in the Vessel Density Region of ROI.

Mean Velocity: Sum of Vessel Velocity in ROI/Pixel Count in the Blood Flow Velocity Region of ROI.

Perfusion index: Mean Velocity in ROI* Vessel ratio in ROI.

### Statistical analysis

SPSS (version 22.0; IBM, NY, USA) was used to perform data analysis, statistical analysis, and the identification of risk factors.

**Figure 4:**
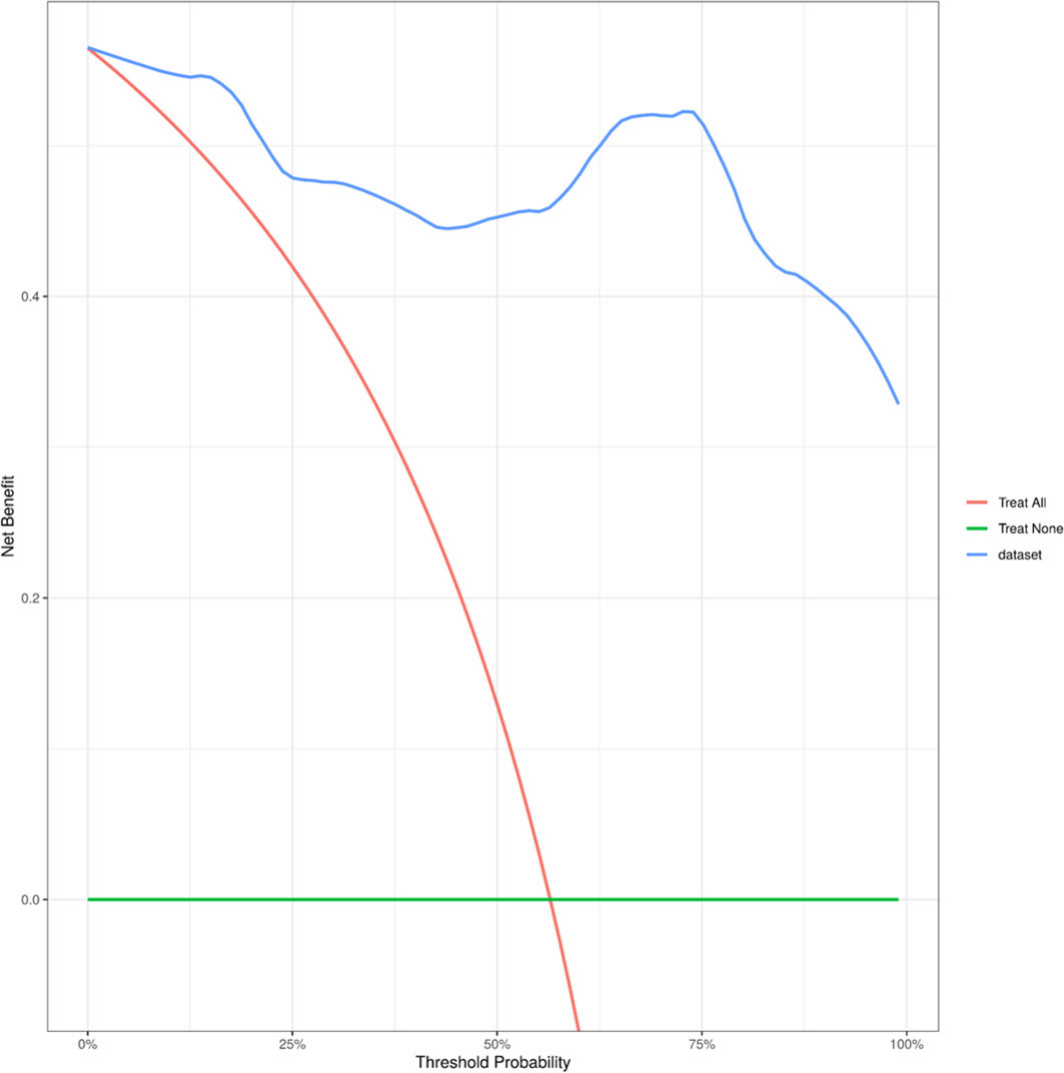
Decision curve analysis showing the net benefit of the model across different threshold probabilities.

**Figure 5:**
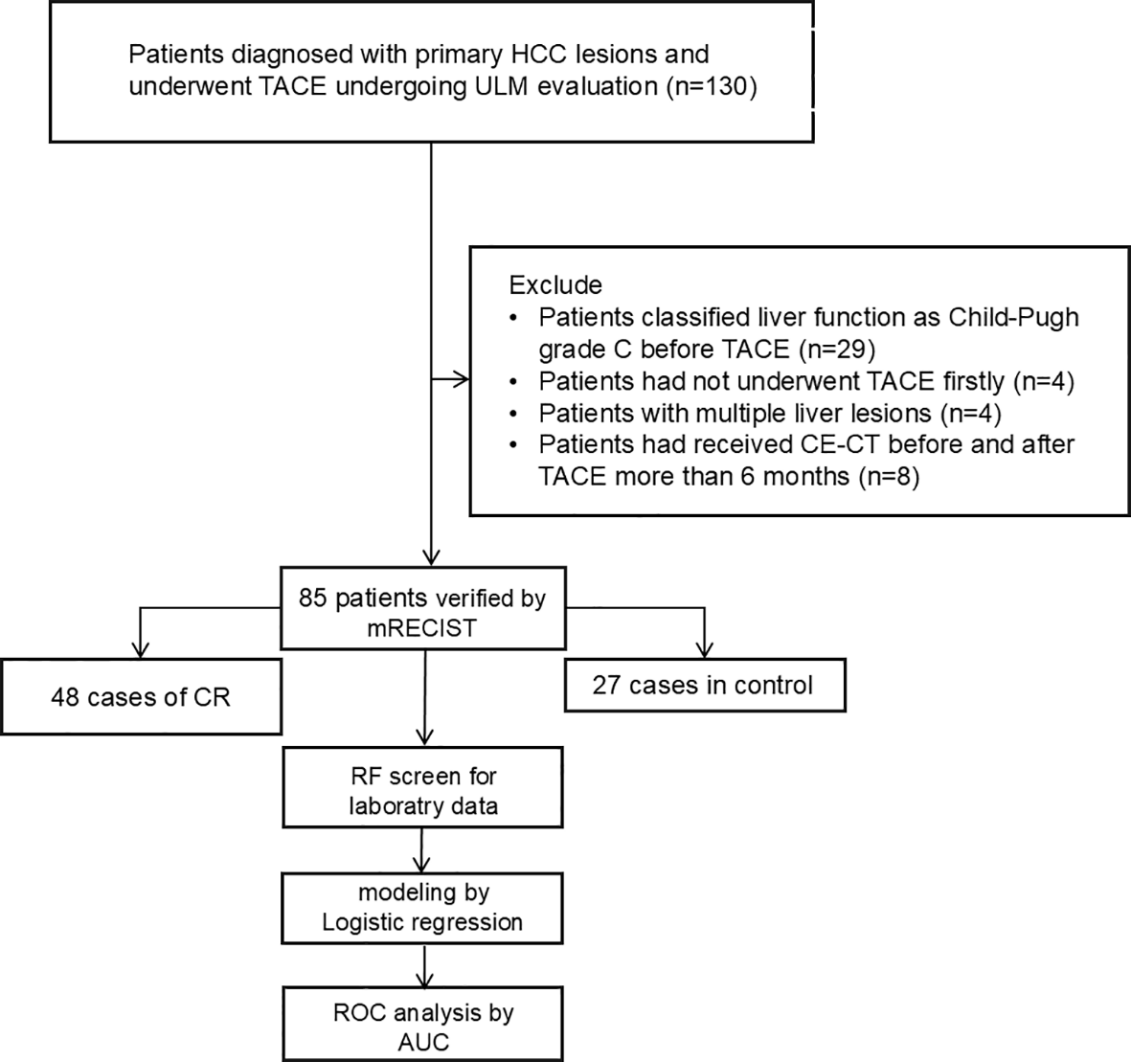
Flowchart of the patient selection process.

The risk factors were screened using Random Forest (RF) regression from raw laboratory data, and missing values were omitted before processing. The selected variables and baseline patient characteristics were selected as candidate parameters. Risk variable selection was calculated using stepwise logistic regression (backward, p<0.05). Adjusted odds ratios (ORs) and the corresponding 95% confidence intervals (95% CIs) were calculated. The receiver operating characteristic (ROC) curve and AUC were estimated for discrimination assessment. Calibration curves were plotted with 1000 bootstrap resamples. (**Figure 4**) The agreement between the predicted probability and the observed outcome was assessed by the Hosmer-Lemeshow test. Furthermore, Decision Curve Analysis (DCA) was performed to quantify the net benefit of the combined model across different threshold probabilities, thereby evaluating its clinical utility (**Figure 5**).

## Results

### Patient characteristics

A total of 85 patients were enrolled in this study. According to the mRECIST criteria, 48 patients were classified into the effective group (CR/PR), and 37 patients were classified into the invalid group (SD/PD). There were no significant differences in baseline demographic data between the two groups (P > 0.05) (**Figure 1**, **Table 2**).

**Table 2:** Clinical features analysis of patient baseline.

Clinical features	CR (n = 48)	Control (n = 37)	χ2 / t / Z	P
Age	60.12 ± 5.64	58.45 ± 6.21	1.294	0.198
Sex (male)	26 (54.17%)	25 (67.57%)	4.052	0.044
Hypertension	6 (12.50%)	7 (18.92%)	0.672	0.412
Diabetesd	4 (8.33%)	5 (13.51%)	0.598	0.439
Size (mm)	14.50 ± 4.12	18.25 ± 5.86	3.468	0.065
location	13 (27.08%)	13 (35.14%)	0.635	0.425

### Correlation analysis by logistic

All parameters were analyzed using binary logistic regression for the identification of risk factors.

**Figure 6:**
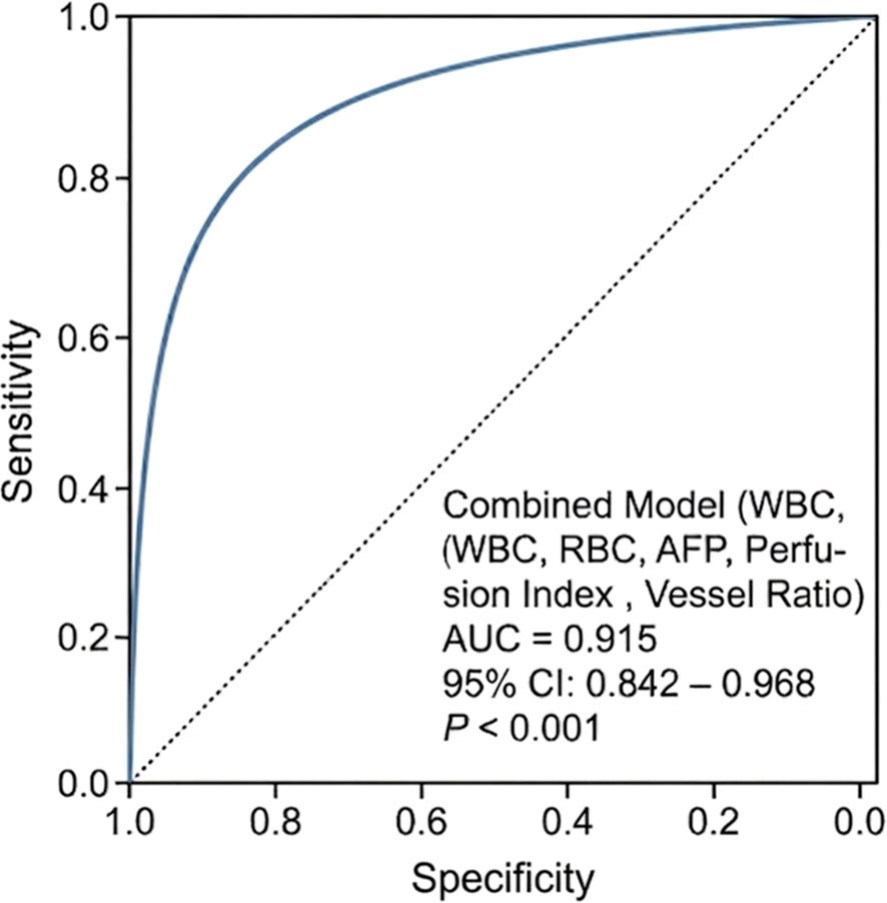
ROC curve for diagnostic efficacy of the high-risk prediction for patients after TACE.

Multivariate logistic regression analysis indicated that ULM-derived vessel ratio, perfusion index, and laboratory indicators including AFP, WBC, RBC, and ALP were independent risk factors for TACE efficacy (all P < 0.05) (**Table 3**, **Figure 6**).

**Table 3:** Logistic regression analysis.

n/N	OR (95% CI)	p value	AUC (95% CI)	Sensitivity	Specificity
WBC	0.45 (0.28 – 0.82)	0.012	0.732 (0.615 – 0.849)	0.685	0.784
RBC	0.38 (0.15 – 0.88)	0.025	0.715 (0.588 – 0.842)	0.702	0.725
ALP	1.05 (1.01 – 1.09)	0.015	0.758 (0.642 – 0.874)	0.81	0.675
Elevated AFP	1.03 (1.01 – 1.06)	0.008	0.785 (0.675 – 0.895)	0.756	0.812
Perfusion Index	4.85 (1.85 – 12.70)	0.002	0.824 (0.726 – 0.922)	0.835	0.76
Vessel ratio	5.12 (2.05 – 13.80)	0.001	0.812 (0.714 – 0.910)	0.815	0.785

## Discussion

HCC imposes a substantial global disease burden, with China accounting for >40% of new cases and annual deaths, and an overall 5-year survival rate of only 14.1%. TACE therapy for HCC currently lacks noninvasive, timely, and effective imaging tools for therapeutic efficacy assessment. This poor prognosis is largely attributed to the high post-TACE recurrence rate, driven by the rich vascular supply of HCC and the ease of formation of collateral circulation.

Conventional modalities such as Doppler ultrasound imaging and CEUS are inhibited by diffraction limits, whereas CE-MRI and CE-CT have shortcomings in real-time quantification. ULM, as a novel CEUS-based technique for visualizing 10–100 μm microvessels, is potentially a new option for post-TACE assessment, but it has not been validated via large-sample clinical assessment.

While one preclinical investigation (Katherine et al.) hypothesized that the ULM could assess early microvascular alterations after TACE, there is no clinical evidence to validate its utility.

In this study, we innovatively applied ULM to evaluate the efficacy of TACE in HCC patients. By combining ULM microvascular parameters with serological markers, we developed a multimodal prediction model. Unlike our preliminary study with a limited sample size, this study expanded the cohort to 85 patients, providing more robust statistical power. The results demonstrated that the combined model achieved an AUC of 0.915, showing excellent diagnostic discrimination performance.

A major concern in radiomics and AI-based studies is the risk of overfitting, particularly with small datasets. To address this, we strictly screened variables using Random Forest and validated the model using calibration curves. The excellent calibration (mean absolute error = 0.076) confirms that our model is reliable and generalizable. Furthermore, the inclusion of ALP and other routine blood indicators (WBC, RBC) alongside AFP enriches the biological plausibility of the model, reflecting both liver function reserve and systemic inflammatory status.

Importantly, ULM overcomes the diffraction limit of conventional ultrasound, enabling clear visualization and quantitative analysis of 10–100 μm microvessels—critical for detecting early residual tumor activity that may be missed by other imaging modalities. Its parameters directly reflect microvascular remodeling in HCC lesions after TACE (a key determinant of tumor viability and recurrence), addressing the limitations of conventional imaging in capturing subtle microvascular changes that signal an early treatment response. As a noninvasive technique, ULM uses microbubble contrast agents and facilitates repeated follow-up, making it more patient-tolerant than CE-CT and more clinically accessible than CE-MRI; additionally, it provides real-time microvascular flow dynamics, offering direct biological insights into the tumor response to TACE and supporting the timely adjustment of treatment strategies.

The perfusion index and ULM-derived vessel ratio jointly reflect tumor cell activity and the vascular support required for tumor survival, which are key indicators of residual tumor viability after TACE. Anemia (decreased hemoglobin) and significant changes in WBC abnormalities (especially an abnormal neutrophil ratio) may be related to tumor activity. It may collectively predict recurrence, as chronic inflammation promotes angiogenesis, and the residual microvasculature sustains tumor regrowth. Decreased WBC and RBC counts indicate tumor remission and reduced inflammation, which may be associated with the tumor after TACE. A significant increase in ALP level (more than twice the normal upper limit) may indicate liver metastasis. According to data from the Chinese Society of Clinical Oncology, approximately 65% of patients with elevated ALP levels are diagnosed with liver metastases on subsequent examinations. AFP is a specific biomarker for HCC after TACE, and its levels usually gradually decrease or return to normal. An increase in AFP suggests the possibility of tumor recurrence or metastasis, which may be caused by post-operative recurrence, worsening inflammation, liver regeneration, and proliferation. The activity of the hepatitis virus may stimulate liver cell synthesis and treatment side effects.

This aligns with contemporary trends in oncology imaging, where multimodal assessment outperforms single-modal tools by capturing both localized structural changes (lesion microvasculature) and systemic physiological status (patient-specific biochemical profiles). In clinical practice, this model enables a more precise identification of high-risk patients, facilitating timely adjustments to the follow-up frequency or initiation of adjuvant therapy.

Beyond statistical significance, clinical applicability is crucial. Previous studies often lacked evidence on whether a new imaging biomarker actually improves decision-making. Our Decision Curve Analysis fills this gap. The results indicate that the combined model provides a higher net benefit than conventional strategies across a broad range of threshold probabilities. This suggests that clinicians can use this model to confidently identify patients who may not respond to TACE early on, allowing for timely adjustment of treatment plans.

The small sample size and single-center design of this study may limit the generalizability of the ULM-related findings and the reliability of the validated parameter cutoffs. However, ULM’s long-term prognostic value and the relative performance of ULM need more practice.

## Conclusion

This study confirms the value of ULM in assessing TACE efficacy for HCC. The combined model, incorporating ULM parameters (vessel ratio, perfusion index) and laboratory markers (AFP, WBC, RBC, ALP), demonstrates high accuracy and robustness. With good calibration and proven clinical net benefit, this non-invasive model offers a promising tool for personalized post-TACE evaluation.

## Data Availability

The raw data supporting the conclusions of this article will be made available by the authors, without undue reservation.
